# Amyloid precursor protein accumulation in glioblastoma is associated with altered synaptic dynamics and immune suppression

**DOI:** 10.1007/s12672-025-03575-z

**Published:** 2025-09-26

**Authors:** Tyrel R. Porter, Mikhail Inyushin, Lilia Kucheryavykh

**Affiliations:** 1 Department of Biochemistry, Universidad Central del Caribe, Bayamón, PR 00956 USA; 2 Department of Physiology, Universidad Central del Caribe, Bayamón, PR 00956 USA

## Abstract

Increased amyloid precursor protein (APP), including both mRNA and protein expression, has been linked to glioblastoma (GBM) yet the physiological effects are poorly understood. In this study, we investigated the relationship between APP protein abundance and mRNA expression in GBM. Gene set enrichment analysis revealed significant positive enrichment of neuron projection and synaptic junction gene sets from Gene Ontology. Additionally, several immune-related processes were negatively enriched, suggesting a broad immune suppression associated with increased presence of APP. These findings suggest a multi-faceted role of APP, linking its presence to modulation of synaptic activity as well as the immune microenvironment in GBM tumors.

**Supplementary Information:**

The online version contains supplementary material available at 10.1007/s12672-025-03575-z.

## Key points

- APP expression in glioblastoma is significantly associated with the upregulation of several gene sets found within neuron projection and synaptic junction.
- Enrichment of monoatomic channel complexes and synaptic components in glioblastoma highlights potential involvement of APP in modulating tumor progression through increased neuronal excitability.
- Gene set enrichment analysis reveals a strong correlation between APP protein abundance and the suppression of immune-related processes, including both innate and adaptive immune responses.

**Supplementary Information:**

The online version contains supplementary material available at 10.1007/s12672-025-03575-z.

## Introduction

GBM is a highly aggressive brain tumor characterized by invasive growth and resistance to treatment, resulting in poor patient outcomes. Previously, amyloid beta (Aβ) accumulation within GBM tumors was identified, with deposits found in both tumor cells and the extracellular matrix [1]. This observation suggests a potential link between amyloid and GBM development, providing rationale for focusing on APP as a factor in GBM progression. It has been shown that APP is highly expressed in GBM when compared to controls in both GBM tumor biopsies as well as cultured GBM cell lines, suggesting an important role in tumor proliferation [2]. Recent data report that APP is expressed in 50% of GBM tumors, with 17% of cases showing expression in more than 50% of the tumor cells, and 89% of cases showing APP expression in tumor-adjacent neurons in more than 50% of cells [3]. Furthermore, research has demonstrated APP’s involvement in driving tumor growth and invasion in breast cancer, providing reason that APP may exert similar pro-tumorigenic effects in GBM [4]. Accordingly, this study investigates how APP protein accumulation in GBM correlates with large-scale transcriptional changes, aiming to uncover pathway-level associations that may influence tumor progression.

## Methods

The relationship between APP protein expression in GBM and mRNA expression in tumor samples was first investigated by gene set enrichment analysis (GSEA) of Gene Ontology gene sets. mRNA expression and protein abundance data from GBM tissue samples were obtained via cBioPortal for Cancer Genomics (CPTAC, Cell 2021) from datasets originally published by Wang et al. (*Cancer Cell* 2021) [5–8]. Downloaded expression data was derived from RNA-Seq analyses and normalized by FPKM-UQ; protein abundance ratios were obtained from mass spectrometry, with methods and calculations described in the original publication [8]. No additional normalization or transformation was applied to the downloaded data.

Correlations between mRNA expression and APP protein abundance ratios were subsequently calculated using Spearman’s rank correlation coefficient across 99 GBM samples and 29,455 genes. Following these calculations, GSEA (v4.3.3) was then performed using a preranked gene list based on Spearman correlation coefficients. Targeted analysis focused on Gene Ontology gene sets accessed from MSigDB (2024.1) [9–11]. Default parameters and weighted enrichment were used with scoring size filters set to a minimum of 15 and a maximum of 500 genes per set. For these analyses, 1,000 permutations were performed, and the data was run without collapsing genes. Gene sets were deemed significant if they had a normalized enrichment score (NES) of ± 1.6, a nominal p-value < 0.05, and a false discovery rate (FDR) < 0.05. However, to support more focused network visualization and interpretation, statistical thresholds were selectively adjusted to a more stringent threshold depending on the enrichment pattern of interest.

Network-based visualizations using enriched gene sets were generated using Cytoscape (v3.10.3) with the Enrichment Map plugin (v3.5.0) [12, 13]. Edges between gene sets were defined based on Jaccard similarity, with a Jaccard coefficient cutoff of 0.5 used to determine inclusion. Detailed gene membership, leading edge gene lists, and signal strength metrics are provided in the Supplementary Data in addition to heatmaps depicting expression patterns of leading edge genes driving the enrichment signals.

## Results

**Fig. 1:**
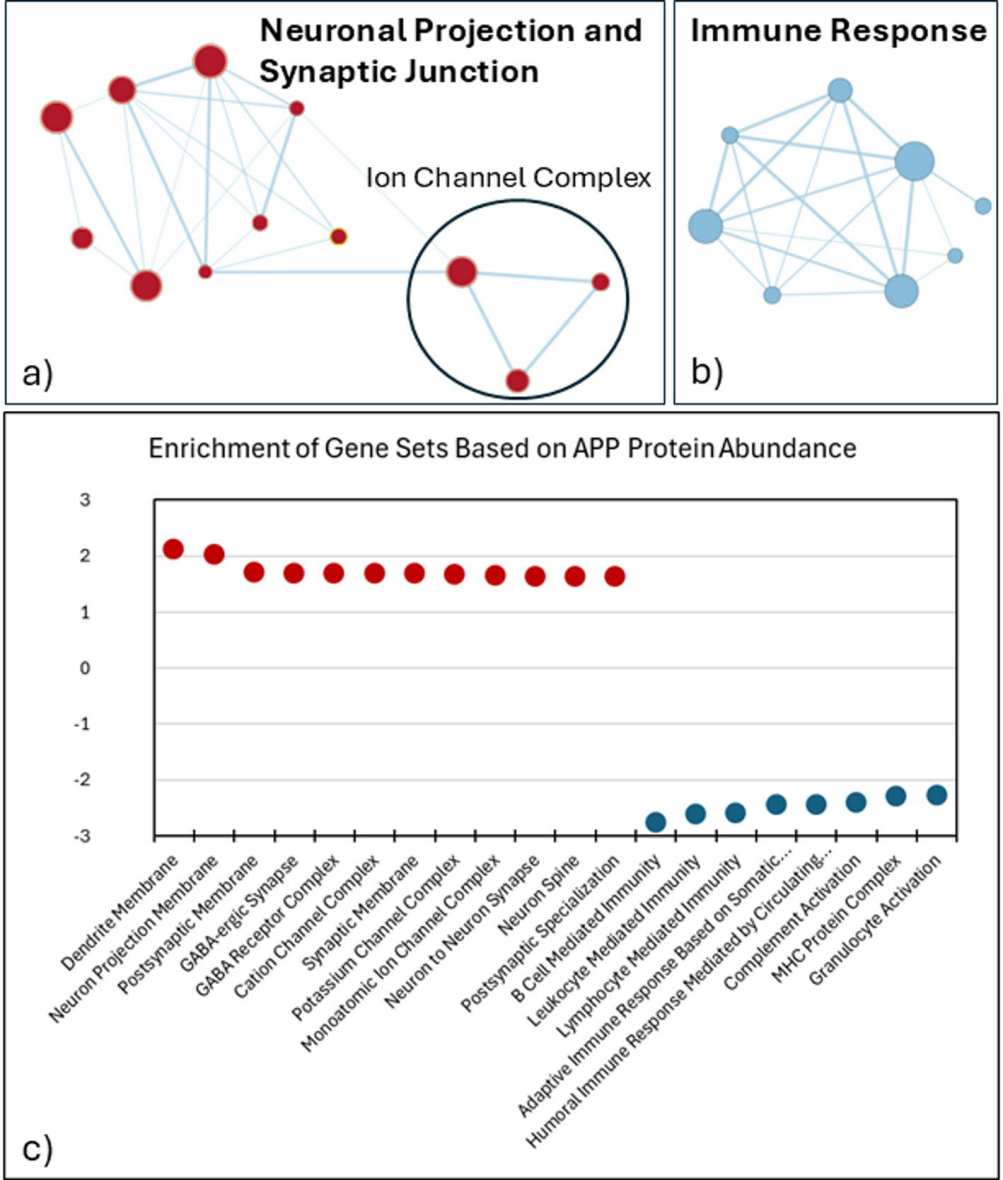
Network containing enrichment of neuronal and immune-related gene sets in glioblastoma, according to APP protein abundance as defined by normalized enrichment scores (NES) (FDR < 0.05, NES > ± 1.6). (a) Enrichment analysis, rendering an enrichment map which reveals overlaps of enriched pathways as a network, of cellular component gene sets demonstrates positive correlations between APP protein abundance and neuron projection and synaptic junction-related processes, and ion channel complexes. (b) Enrichment map including both Cellular Component and Biological Process gene sets, highlighting negative correlations between APP abundance and the suppression of immune-related processes. (c) Dot plot summarizing NES appearing in enrichment maps for Neuron Projection and Synaptic Junction in addition to Immune Response, which include significantly enriched and suppressed gene sets, respectively

GSEA of Gene Ontology Cellular Component (GOCC) gene sets yielded 494 usable gene sets following size-based filtering. Applying significance thresholds, 133 gene sets were identified as significantly enriched, of which 106 showed negative and 27 showed positive NES. To refine the network visualization and focus on the most robust associations, we applied an additional stringency filter of nominal p-value < 0.005 prior to enrichment map visualization. The enrichment map revealed several distinct network components, a strongly enriched group containing neuron projection gene sets (GO:0043005) was observed, including dendrite membrane (NES = 2.11; 28 of 40 genes in leading edge), neuron projection membrane (NES = 2.02; 37 of 60), and neuron spine (NES = 1.63; 69 of 174) Fig. 1a and c. Within this cluster, positive enrichment was observed in synaptic junction gene sets (GO:0045202), including postsynaptic membrane (NES = 1.70), GABA receptor complex (NES = 1.68; 12 of 21 genes), GABA-ergic synapse (NES = 1.68; 39 of 79), synaptic membrane (NES = 1.68; 171 of 400), neuron to neuron synapse (NES = 1.63; 146 of 367), and postsynaptic specialization (NES = 1.63; 115 of 352). Further subdivision revealed gene sets related to monoatomic ion channel complexes (GO:0034702, NES = 1.65; 124 of 340 genes), including both cation channel complex (NES = 1.68; 79 of 203) and potassium channel complex (NES = 1.67; 42 of 100). [Supplementary Data 1,Supplementary Fig. 1].

Given that the majority of significantly enriched GOCC gene sets exhibited negative NES, we performed a complementary enrichment analysis to further investigate the biological processes associated with negatively correlated gene expression patterns to more comprehensively characterize these negatively associated gene expression patterns. Including Gene Ontology Biological Processes (GOBP) in addition to GOCC categories and initially identified 171 gene sets that met initial significance criteria (nominal p-value < 0.05, FDR < 0.05). Considering the addition of more gene sets, a more rigorous statistical threshold (nominal p-value < 0.001, FDR < 0.001) was implemented prior to gene set enrichment network analysis. This approach was intended to minimize false positives and highlight only the most robust and biologically meaningful associations among downregulated pathways. The resulting enrichment map revealed a distinct cluster of negatively enriched immune response gene sets, suggesting that APP expression in GBM is linked to suppression of immune activity. More specifically, the resulting enrichment map revealed a negatively enriched cluster of immune response gene sets (GO:0006955), including B cell-mediated immunity (NES = -2.77; 111 of 188 genes in leading edge), leukocyte-mediated immunity (NES = -2.61; 228 of 450), lymphocyte mediated immunity (NES = -2.60; 182 of 353), and complement activation (NES = -2.42; 34 of 64) [Fig. 1b and c]. Additional suppressed processes included granulocyte activation (NES = -2.27; 31 of 49), adaptive immune response based on somatic recombination of immune receptors (NES = -2.45; 189 of 364), humoral immune response mediated by circulating immunoglobulin (NES = -2.44; 35 of 55), and MHC protein complex (NES = -2.29; 21 of 24) [Supplementary Data 2, Supplementary Fig. 2].

## Discussion

### Neuronal remodeling and tumor invasion

Our results showed transcriptional increases in neuron projection, synaptic junction, particularly within ion channels. Interestingly, increased synaptic activity and neuronal remodeling has been linked with GBM progression, as well as higher mortality [14, 15]. More specifically, synaptic activity shows mechanistic links which facilitate glioma proliferation, in part through the amplification of depolarizing currents in tumor cells. Importantly, Kv7 channels are key regulators of neuronal excitability and synaptic stability. This aligns with emerging evidence that glioma cells create positive feedback via neuronal circuits to promote their own growth [16]. In our study, the observed positive enrichment of potassium channel complex gene sets, particularly Kv7 subunits, raises the possibility that APP accumulation contributes to the electrophysiological landscape that enables this tumor excitability, although further mechanistic research would be required to confirm this assumption.

With this in mind, transient axonal glycoprotein-1, a known APP ligand, has been shown to induce apoptosis-associated gene expression in glioma cells without triggering apoptosis, instead promoting proliferation [17]. This paradoxical effect reflects the broader complexity of APP-related pathways in neural tumors, where proteins canonically associated with neurodegeneration may paradoxically support tumor aggressiveness.

### Immunosuppressive microenvironment

In addition to altered synaptic dynamics, our results show APP appears to play a key role in modulating the immunosuppressive microenvironment in GBM. Previous studies have shown that APP and its cleaved products, such as Aβ, regulate immune responses, particularly affecting macrophages by reducing TNFα and IL-6 secretion and increasing IL-10, promoting an anti-inflammatory state [18]. Beyond its effects in GBM, APP has also been shown to play a critical role in tumor growth and dispersal in brain metastasis [2]. For instance, silencing of APP in melanoma cells significantly reduced brain metastasis and metastatic burden in mice, implicating APP in promoting brain colonization [19]. Considering our results indicated suppression of B cell-mediated immunity, leukocyte-mediated immunity, and complement activation, in addition to these mechanisms, may suggest that APP helps tumor cells evade immune detection by downregulating both adaptive and innate immune responses, including macrophages, B cells, and microglia.

These results raise some interesting questions, considering one study linked APP to the upregulation of pro-inflammatory markers, specifically IL-1β, COX-2, and cPLA2 in GBM [2]. In contrast, our findings suggest that higher APP abundance coincides with transcriptional suppression of immune-related pathways. This may reflect a shift from early tumor-associated inflammation toward a more immune-evasive state, potentially driven by neuroimmune interactions. It is also possible that these observations reflect a context-dependent role for APP which promotes local inflammation early in tumor development while later facilitating immune evasion through suppression of adaptive immune responses. Moreover, recent evidence suggests that IL-1β, despite being a classical pro-inflammatory cytokine, can also contribute to immune suppression within the tumor microenvironment independently of inflammasome activation [20]. This dual function is supported by prior evidence that APP and its cleavage products shift macrophages toward an anti-inflammatory phenotype, suggesting APP may contribute to both inflammatory signaling and immunosuppression in GBM [18].

### Limitations

While our findings demonstrate significant associations between APP protein abundance and coordinated transcriptional changes in GBM, several limitations should be noted. First, this study is based on correlation analyses, which cannot establish causal relationships. Although GSEA provides robust pathway-level enrichment, functional studies using APP knockdown or overexpression in GBM models are needed to confirm its mechanistic role in modulating synaptic and immune processes. Furthermore, the transcriptomic and proteomic data analyzed were derived from bulk tumor tissue, which includes a mixture of tumor cells, neurons, glia, and immune cells. As such, observed associations with APP may partly reflect expression patterns in surrounding stromal or neural cells. Future studies using single-cell or spatial transcriptomic approaches would allow for more precise resolution of cell-type-specific expression and signaling.

Lastly, while our pathway-level analysis identified coherent enrichment patterns, further investigation into individual genes within leading edge subsets may help clarify which specific genes are most strongly associated with APP expression and whether they have functional roles in GBM progression or immune modulation and subsequent effects on clinical outcomes.

## Conclusion

This study identified significant correlations between APP protein abundance and gene expression patterns associated with both increased neuronal excitability and suppression of immune-related pathways in GBM. These findings suggest that APP may serve as a molecular bridge between neuronal remodeling and immune evasion, processes that are increasingly recognized as key drivers of glioma progression. While our results do not directly demonstrate functional consequences such as tumor proliferation or invasion, the observed upregulation of synaptic and ion channel-related genes, alongside downregulation of immune signaling pathways, raises the possibility that APP contributes to a tumor-supportive microenvironment through neuro-immune interactions.

## Supplementary Information

Below is the link to the electronic supplementary material.

Supplementary Material 1

Supplementary Material 2

Supplementary Material 3

Supplementary Material 4

## Data Availability

Data used in this publication were generated by the National Cancer Institute Clinical Proteomic Tumor Analysis Consortium (CPTAC) and accessed via cBioPortal for Cancer Genomics (https://www.cbioportal.org/study/summary? id=gbm_cptac_2021). The datasets analyzed in this study were originally published in: Wang LB, Karpova A, Gritsenko MA, et al. Proteogenomic and metabolomic characterization of human glioblastoma. *Cancer Cell* . 2021;39(4):509-528.e20. doi:10.1016/j.ccell.2021.01.006.

## Declarations

### Research involving human or animal participants

This study did not involve direct experimentation on humans or animals.
