# Supplementary figures and images for: Amyloid precursor protein accumulation in glioblastoma is associated with altered synaptic dynamics and immune suppression
Source: Discov Oncol. 2025 Sep 26;16:1730. doi: 10.1007/s12672-025-03575-z (PMC12474803; doi:10.1007/s12672-025-03575-z)

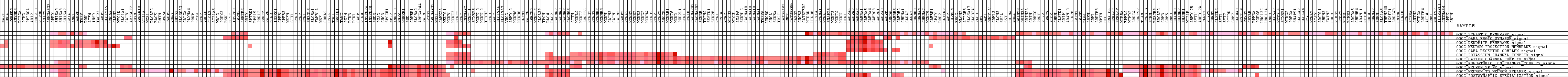

Supplement: Supplementary file 3 — Supplementary Material 3 [file 12672_2025_3575_MOESM3_ESM.png]

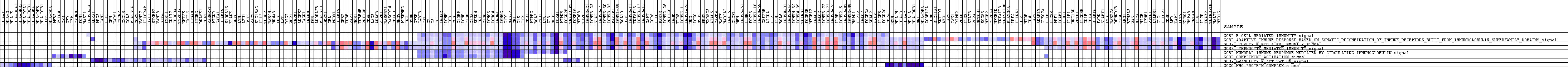

Supplement: Supplementary file 4 — Supplementary Material 4 [file 12672_2025_3575_MOESM4_ESM.png]
